# Clinical scale rapid expansion of lymphocytes for adoptive cell transfer therapy in the WAVE^® ^bioreactor

**DOI:** 10.1186/1479-5876-10-69

**Published:** 2012-04-04

**Authors:** Robert PT Somerville, Laura Devillier, Maria R Parkhurst, Steven A Rosenberg, Mark E Dudley

**Affiliations:** 1 Surgery Branch, National Cancer Institute, National Institutes of Health, Bethesda, MD, USA

**Keywords:** Human, T Cells, Tumor Immunity, T Cell Receptors, Adoptive Immunotherapy

## Abstract

**Background:**

To simplify clinical scale lymphocyte expansions, we investigated the use of the WAVE^®^, a closed system bioreactor that utilizes active perfusion to generate high cell numbers in minimal volumes.

**Methods:**

We have developed an optimized rapid expansion protocol for the WAVE bioreactor that produces clinically relevant numbers of cells for our adoptive cell transfer clinical protocols.

**Results:**

TIL and genetically modified PBL were rapidly expanded to clinically relevant scales in both static bags and the WAVE bioreactor. Both bioreactors produced comparable numbers of cells; however the cultures generated in the WAVE bioreactor had a higher percentage of CD4+ cells and had a less activated phenotype.

**Conclusions:**

The WAVE bioreactor simplifies the process of rapidly expanding tumor reactive lymphocytes under GMP conditions, and provides an alternate approach to cell generation for ACT protocols.

## Background

Metastatic melanoma is a highly aggressive cancer with a 5 year survival rate of less than 10% [1]. The FDA currently approves three treatments for metastatic melanoma, dacarbazine [2]), interleukin-2 [3], and ipilimumab [4]. Dacarbazine has an objective response rate of 10-15% with few, if any, durable remissions [2]. IL-2 has an objective response rate of about 15%, with a complete response of less than 5% [2,5]. In a large randomized trial with ipilimumab, the objective response rate was reported to be about 10%, with 0.6% complete responses [4]. Inhibitors of dominant BRAF mutations are currently in large, randomized clinical trials, and could be approved in the near future [6]. Phase II studies with these agents did not result in a durable complete response for most patients and so additional approaches to treating advanced melanoma are required.

In light of this, the development of adoptive cell transfer (ACT) therapy using autologous tumor infiltrating lymphocytes (TIL) or peripheral blood lymphocytes (PBL) modified to express specific T cell receptors targeting tumor antigens, holds great promise [7]. The adoptive transfer of TIL combined with a lymphodepleting preconditioning regimen can yield objective responses in 48-70% of metastatic melanoma patients, with up to 40% of patients experiencing a durable complete response [8-10].

It is not always possible to generate a TIL culture from every resected tumor and for most tumor histologies there are few, if any, TIL that are able to recognize autologous tumor *in vitro*. To increase the range of tumors that can be treated by this approach, it is possible to genetically engineer peripheral T cells to express T cell receptors or chimeric antigen receptors that target specific tumor antigens [11]. A recent report described the use of NY-ESO-1 transduced PBL following lymphodepletion [12]. Five of 11 patients with advanced refractory melanoma demonstrated objective clinical responses to treatment, and four of six patients with refractory synovial sarcoma exhibited objective tumor regression. Objective clinical responses with genetically retargeted lymphocytes have also been reported in lymphoma and colorectal carcinoma [13,14].

These adoptive transfer therapies rely on the large scale *ex vivo *activation and expansion of TIL or genetically modified PBL generating an average of about 5 × 10^10 ^cells for reinfusion into patients. Traditionally this has been achieved by static culture methods. In our facility, the final "rapid expansion protocol" (REP) phase of cell expansion requires two weeks of culture and is initiated in multiple T175 flasks. After sufficient expansion cells are transferred to 3 liter gas permeable bags. Up to 24 bags are required, along with frequent culture manipulations for accurate counting, and to allow episodic batch feeding and bag splitting for maintenance of cell densities in the optimal cell concentration range (0.5-1.5 × 10^6 ^cells per ml), while diluting waste products such as ammonia and lactate. The semi-open system coupled with frequent culture manipulations introduces multiple opportunities for contamination, thus, highly skilled personnel are required. In addition the final cell product volume can be as high as 50 liters, which requires specialized equipment, disposables, dedicated space and substantial harvest time. These technical challenges have proved an impediment to a wider dissemination of ACT.

We and others have investigated alternate systems for lymphocyte expansion for individualized patient therapies. An improved TIL expansion process would optimally be simplified, "closed," capable of producing the desired number of cells in a minimal volume, and affordable. In this paper we investigate the use of the WAVE bioreactor, a system that utilizes continuous media exchange, to rapidly expand tumor infiltrating lymphocytes and genetically modified PBL under GMP conditions for clinical trials. Our studies demonstrate the WAVE system is capable of generating TIL and PBL gene modified cells with comparable properties to 3 liter gas permeable static bags. Cellular and immunological analysis suggests that the WAVE bioreactor may be a preferred method of cell expansion for some cell subsets or phenotypes.

## Methods

### Patient material, tumor cell lines, and retroviral vectors

All excised tumor tissue and peripheral blood products were collected as part of approved clinical protocols (Clinical Research Center Institutional Review Board approved) and all patients gave their signed informed consent.

Tumor cell lines 624mel (HLA-A2^+^) and 888mel (HLA-A2^-^), and the TAP deficient cell line T2 were maintained in RPMI1640 supplemented with 10% heat inactivated calf serum (Hyclone, Logan UT), 12 mM L-glutamine, 25 mM HEPES, 55 μM β-mercaptoethanol, 100 units/ml penicillin, 100 μg/ml streptomycin, and 10 μg/ml gentamicin.

γ-retroviral vectors used to transduce patient PBL were manufactured for ongoing clinical trials in accordance with current good manufacturing practices by the Indiana University Vector Production Facility or the Surgery Branch Vector Production Facility. T cell receptors (TCR) used in this study targeted one of the following epitopes (all restricted by HLA-A2): NY-ESO-1:157-165, MART-1:27-35, or gp100:154-162.

### Initiation of young TIL and genetically modified peripheral blood lymphocytes

Young TIL cultures were generated as previously described [15-17]. Briefly, homogenous cell suspensions were established from excised tumors using overnight enzymatic digestion or by a GentleMACS dissociator (Miltenyi Biotec). Cell number and viability were determined by trypan blue staining and counting on a Neubauer hemocytometer. The cell suspensions were plated in 24 well plates at a concentration of 0.5 × 10^6 ^cells per ml in RPMI1640 supplemented with 10% heat inactivated human AB serum, 12 mM L-glutamine, 25 mM HEPES, 55 μM β-mercaptoethanol, 100 units/ml penicillin, 100 μg/ml streptomycin, 10 μg/ml gentamicin, and 6000 IU/ml IL-2. The cells were incubated at 37°C with 5% CO_2_. On day 5 after initiation of the TIL culture half the media was replaced with fresh media. Media was then exchanged and confluent wells were split every 2-3 days, and when a sufficient number of cells were obtained, all wells were pooled and rapid expansions were initiated. Some young TIL underwent CD8+ enrichment using GMP quality immunomagnetic beads (Miltenyi) as previously described prior to rapid expansion.

Patients PBL were transduced as previously described [12,18,19]. Briefly, BPL obtained by apheresis were, purified over a ficoll-Hypaque cushion and stimulated with 50 ng/ml anti-CD3 (OKT3) and 300 IU/ml IL-2 in AIM V supplemented with 5% heat inactivated human AB serum. After 2 days the cells were harvested, washed, and transduced by plating in 6-well plates that were pre-coated with retrovirus and Retronectin^®^. The cells were incubated at 37°C overnight, and then transferred to a second set of plates for a second transduction. After overnight incubation, the transduced PBMC were washed, resuspended, and maintained at 0.5-2 x10^6 ^cells per ml in AIM V media supplemented with 5% heat inactivated human AB serum plus 300 IU/ml IL-2.

### Initiation of rapid expansion protocol (REP)

The REP was initiated in T175 flasks for the first seven days as previously described. Briefly, TIL (1 × 10^6^) or transduced PBL (2 × 10^6^) were mixed with a 200 or 100 fold excess (respectively) of irradiated (40 Gy) feeder cells, 30 ng/ml OKT3 anti-CD3 antibody, and 3000 IU/ml IL-2 in 150 ml media. Five days later, about 2/3 of the media was removed by aspiration and replaced with fresh media containing 3000 IU/ml IL-2. Two days later, on day 7 of the REP, cells were transferred from flasks to an alternate growth chamber as described below.

### Rapid expansion using gas permeable static bags

On day 7 of the REP, the cells and 300 ml media from two T175 flasks were transferred to a 3 liter Baxter LifeCell culture bag, and an equal volume of media (AIM V supplemented with 5% human serum plus 3000 IU/ml IL-2) was added. Bags were sampled and the cell concentration was determined daily. The bags were fed as required to maintain a cell density of approximately 0.5-1.5 × 10^6 ^cells/ml. Each patient's cells were initially fed with up to 10 liters of AIM V supplemented with 5% human AB serum and 3000 IU/ml IL-2. Subsequent AIM V (without serum) supplemented with 3000 IU/ml of IL-2 was used to feed cells. Cell products, including TIL and TCR transduced PBL, with a target number at infusion of 5 × 10^10 ^cells would start from 36-48 T175 flasks and have a final process volume at the conclusion of the REP (day 14) of 30 - 50 liters.

### Rapid expansion using the WAVE bioreactor

On day 7 of the REP a 10 liter Cellbag was attached to the tray of a WAVE bioreactor 2/10 system. The Cellbag was inflated with 5% CO_2 _and the system was tared. A bag of medium and a 20 liter bag to collect waste were attached to the appropriate ports of the Cellbag. Media was added to the cell bag to a final volume of 1.5 liters. The media was warmed to 37°C and aerated by rocking at 7 rpm at an angle of 6° for 2 hours. TIL or genetically modified PBL were introduced to the CellBag by gravity feed and the volume was made to 3 liters. Media was perfused in 50 ml increments using a semicontinuous program. Samples were sterilely drawn daily from the needleless ports for determination of cell number, lactate, and glucose concentrations. The glucose concentration in the cell bag was measured using an Accu-Chek hand held blood glucose monitor and test strips. Lactate levels were measured using the Lactate Pro™ blood lactate test meter from Arkray (Kyoto, Japan). Perfusion rates were varied daily to maintain a media glucose concentration of approximately 170 mg/dl. Initially cells were perfused with AIM V supplemented with 5% human AB serum, 0.02% Pluronic, and 3000 IU/ml IL-2 (10 liters), after the initial 10 liters of media were expended cells were perfused with AIM V (without serum) supplemented with 0.02% Pluronic and 3000 IU/ml of IL-2. All media bags were maintained at 4°C in the dark to reduce degradation of components.

### Safety, functional, and phenotypic analysis

Cell products were tested for contamination with mycoplasma, fungi and aerobic or anaerobic bacteria by the Clinical Center hospital Clinical Laboratory Improvement Amendments (CLIA)-approved microbiology laboratories. Endotoxin was quantified using the FDA approved Pyrogent 5000 kinetic turbidimetric assay from Lonza. Quantification of tumor cell contamination in expanded TIL cultures was performed using HMB45 and anti-MART-1 antibodies with a validated assay in the Clinical Center Department of Cytopthology.

The expression of the following cell surface markers on TIL and genetically modified PBL expanded in LifeCell bags and the WAVE bioreactor were compared by 6 color flow cytometry on a Canto II Flow cytometer: CD3, CD4, CD8, CD56, CD45RA, CD45RO, CD62L, CCR7, CD25, CD27, CD28 (BD Biosciences), In addition, for the genetically modified PBL, the expression of the introduced TCR was analyzed by staining with the cognate tetramer complex (Beckman).

Antigen specific cytokine secretion by TCR transduced PBL was measured as previously described [17]. Briefly, genetically modified PBL were incubated with HLA-matched or HLA-mismatched melanoma cell lines or T2 cells pulsed with cognate or non-specific peptides overnight at 37°C, and the IFN-γ concentration of coculture supernatants was quantified by an enzyme linked immunosorbent assay. A positive signal was defined as twice the amount of IFN-γ released by the relevant negative control well(s) and at least 200 pg/ml.

### Statistical analysis

All data was analyzed using the statistical analysis tools in Excel, including Student's *T*-Test and Paired *T*-Test as indicated in the text. Results are expressed as the mean ± the standard error.

## Results

### Active perfusion of the WAVE bioreactor creates A stable culture environment

**Figure 1:**
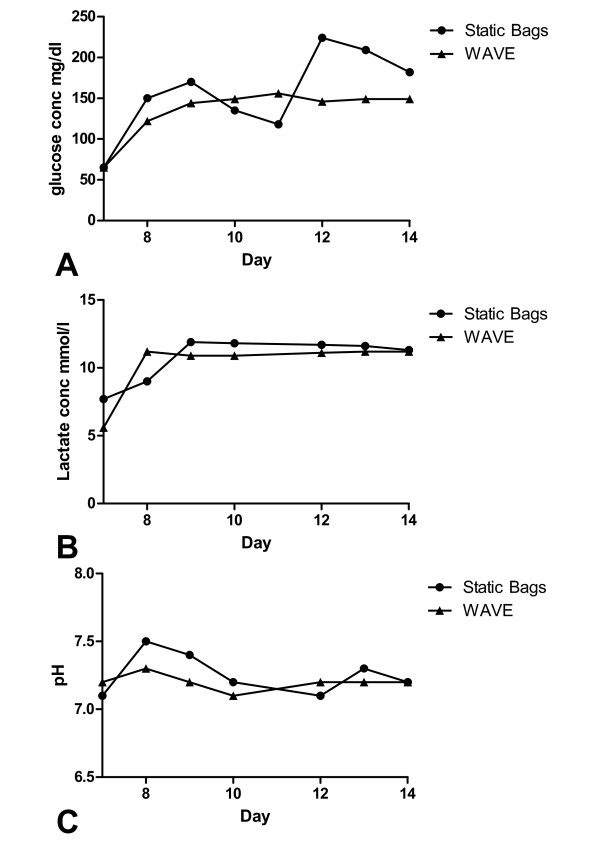
**The Wave bioreactor creates a more stable culture environment for rapidly expanding lymphocytes**. A comparison of media depletion for a culture grown in static bags (circles) or the WAVE bioreactor (triangles). a) glucose concentration. b) lactate concentration. c) pH.

We wanted to determine if the culture environment of lymphocytes rapidly expanded in the WAVE bioreactor, with its active perfusion of media, was more stable than that of lymphocytes undergoing rapid expansion in static bags with an episodic feeding regimen. Patients' cells were simultaneously rapidly expanded in static bags and the WAVE bioreactor and the glucose and lactate concentration, and pH of the media was monitored daily throughout the duration of the expansion. The concentration of glucose and lactate, and the pH of the media was measured prior to adding the cells to the two bioreactors on day 7. Figure 1 shows the data from a representative expansion. The glucose concentration showed the greatest degree of variation between the two bioreactors, with the WAVE bioreactor stabilizing at about 150 mg/dl within two days of inoculation and perfusion initiation, while the glucose concentration in static bags peaked with each addition of media before undergoing a gradual decline (Figure 1a). The lactate level (Figure 1b) stabilized at approximately 11 mmol/l within 24 hours of cell addition and perfusion initiation in the WAVE bioreactor, while the static bags took longer to stabilize. By the end of the rapid expansion the lactate levels in both bioreactors converged. The pH in both bioreactors fluctuated within a narrow range (Figure 1c); however the degree of fluctuation was smaller in the WAVE bioreactor. In comparison to the static culture bags, the WAVE bioreactor and its active perfusion of media creates a more stable culture environment throughout the duration of the rapid expansion.

### Clinical scale rapid expansions of TIL and genetically modified PBL can be achieved in the WAVE bioreactor

**Figure 2:**
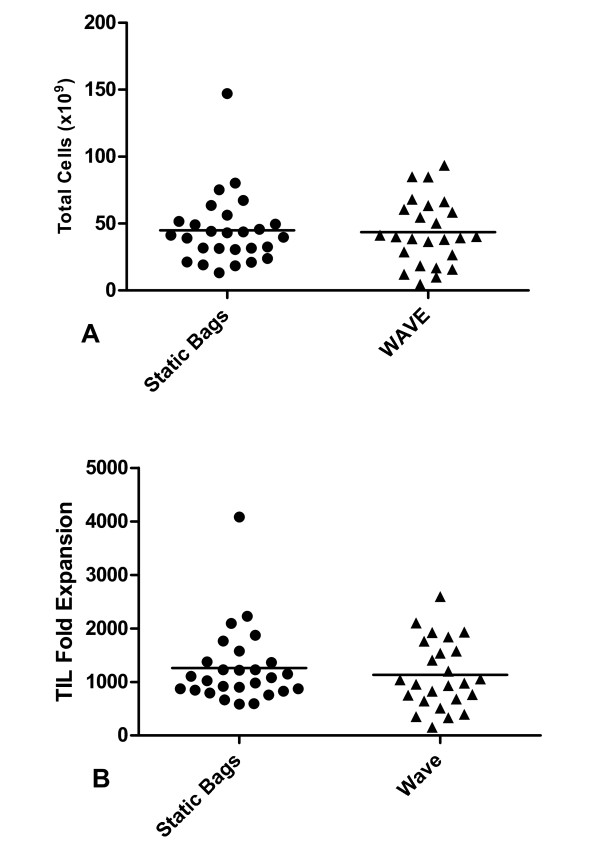
**The WAVE bioreactor and static bags expand T lymphocytes comparably**. A comparison of TIL expanded in either static bags (n = 25, circles) or the WAVE bioreactor (n = 27, triangles). a) Total TIL number expanded b) Fold expansion.

TIL and genetically modified PBL can be rapidly expanded in the WAVE bioreactor to clinically useful numbers. Patients either had their TIL expanded solely in the Static bags (n = 25) or solely in the WAVE bioreactor (n = 27). The mean of total cell numbers harvested from static bags was 4.5 × 10^10 ^(± 5.1 × 10^9^) and 4.4 × 10^10 ^(± 4.9 × 10^9^) for WAVE bioreactor (Figure 2a). This corresponds to a mean fold expansion of 1259 (± 137) and 1130 (± 127) for static bags and the WAVE bioreactor respectively (Figure 2b). There was no statistical difference in the fold expansions achieved in the two bioreactors. The viability of TIL expanded in static bags and the WAVE bioreactor was also similar, 96.95% and 95.26% respectively (p = 0.07). We were concerned that differences in the ability of the bioreactors to expand cells maybe masked by the fact we were analyzing different populations of cells.

**Figure 3:**
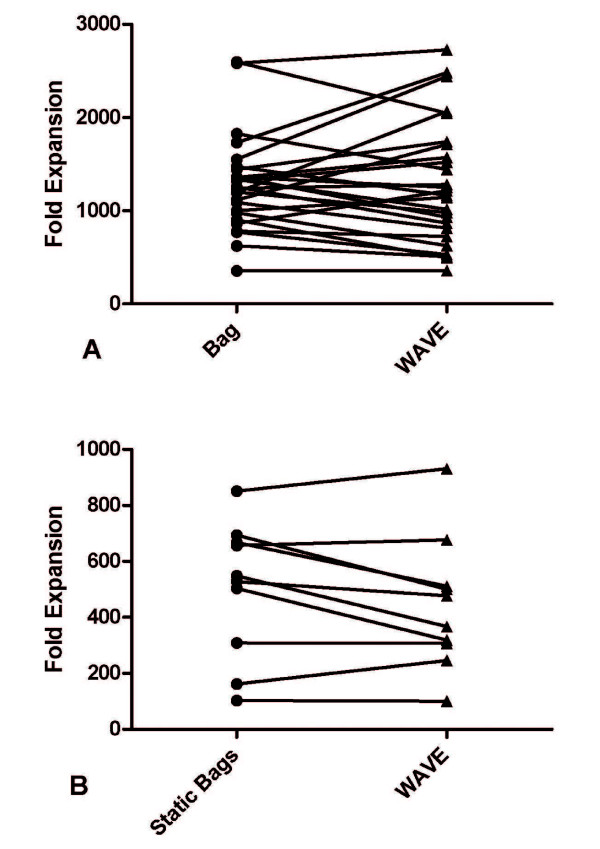
**The WAVE bioreactor and static bags expand the same lymphocyte cultures comparably**. a) A comparison of the fold expansion of TIL that underwent concurrent rapid expansion in static bags (circles) and the WAVE bioreactor (triangles). b) A comparison of the fold expansion of genetically modified PBL that underwent concurrent rapid expansion in static bags (circles) and the WAVE bioreactor (triangles).

To address this interpopulation variation we took individual patients (n = 26) and simultaneously expanded some of their cells in static bags and some of their cells in the WAVE bioreactor to determine if comparable numbers of cells and fold expansions could be achieved when analyzing the same populations of cells (Figure 3a). The overall fold expansion of patients' TIL was independent of the bioreactor used for expansion (paired *T*-Test, p = 0.9), with a mean fold expansion of 1281 (± 100) and 1295 (± 128) for static bags and the WAVE bioreactor respectively. TIL expanded from WAVE bioreactors were also active in mediating tumor regressions in patients. 25 patients received TIL expanded in the WAVE bioreactor, of which 6 had a reduction in the diameter of their evaluable lesions of greater than 30%.

Similarly, the WAVE bioreactor was able to expand NY-ESO, MART-1 and gp-100 TCR transduced peripheral blood mononuclear cells (n = 10) to clinically useful levels. Each patient's transduced PBL were simultaneously expanded in static bags and the WAVE bioreactor. There was no significant difference in the overall fold expansion of genetically modified PBL in static bags or the WAVE bioreactor (444 ± 74 and 502 ± 76, p = 0.13) (Figure 3c). Therefore, use of the WAVE bioreactor as the sole bioreactor for cell expansion would yield the same number of TIL and genetically modified PBL as would growth in static bags.

### Wave bioreactor expanded, TCR transduced lymphocytes have A higher apparent avidity than bag expanded lymphocytes

Successful adoptive transfer treatments rely on the generation of large quantities of functionally active cells. For this reason, we assessed the functionality of cells that had under gone concurrent rapid expansions in static bags and the WAVE bioreactor (Table 1). TCR transduced PBL rapidly expanded in the WAVE bioreactor secreted more IFN-γ in response to 0.1, 0.01 and 0.001 μm of cognate peptide pulsed on to T2 cells, when compared with cells expanded in static bags. This observation of increased IFN-γ release was independent of the TCR specificity, suggesting that WAVE bioreactor expanded cells have a higher apparent avidity for their cognate peptide. When TCR transduced PBL from the two bioreactors were cocultured with HLA matched and mismatched melanoma cell lines, comparable amounts of IFN-γ were secreted, indicating that the apparent higher avidity of WAVE bioreactor expanded cells did not translate into enhanced tumor recognition.

**Table 1:** A table of IFN-γ secretion in response to HLA matched and mismatched cell lines and to T2 cell pulsed with peptide recognized by the transduced TCR

	Melanoma Cell Lines				T2 cells pulsed with peptide (μM/ml)					
			**938**	**624**	**Irrelevant Peptide**		**Specific Peptide**			

	**TCR**	**Bioreactor**	**A2-**	**A2+**	**1.0**	**1.0**	**0.1**	**0.01**	**0.001**	**0.0001**

NY-ESO	Patient 1	Static Bags	40	19300	44	> 126650	45600	5095	1080	136
		Wave	68	46950	128	> 182100	83200	23350	2770	646
	
	Patient 2	Static Bags	345	39700	694	> 241000	52250	4195	985	339
		
		Wave	410	40700	1876	> 219650	58400	8650	2374	1302
	
	Patient 3	Static Bags	79	> 31250	84	> 84800	23850	2425	242	40
		
		Wave	78	> 30150	120	> 83000	31500	4830	534	139
	
	Patient 4	Static Bags	16	4725	27	11045	1075	166	45	29
		
		Wave	18	5770	43	12445	1610	236	54	33
	
	Patient 5	Static Bags	7	11780	855	> 113300	25555	2635	1346	14
		
		Wave	1201	16140	147	> 125300	63400	8230	469	177
	
	Patient 6	Static Bags	129	3505	105	9535	1080	147	40	12
		
		Wave	36	4000	99	26950	2560	315	118	80
	
	Patient 7	Static Bags	23	6565	40	24750	3445	818	393	159
		
		Wave	78	3550	290	> 45100	6240	911	441	204

MART-1	Patient 8	Static Bags	98	> 77225	160	> 58200	11480	1375	544	NA
		
		Wave	148	91625	547	62200	20875	3630	1510	NA
	
	Patient 9	Static Bags	12	66500	30	> 180600	93700	370	490	NA
		
		Wave	65	> 162700	197	> 260700	> 154600	3000	2897	NA

gp154	Patient 10	Static Bags	18	32950	22	NA	4360	2600	739	479
		
		Wave	18	42650	55	NA	8075	5290	1785	684

### The phenotype of TIL and genetically modified PBL expanded in static bags and the WAVE bioreactor differ

**Figure 4:**
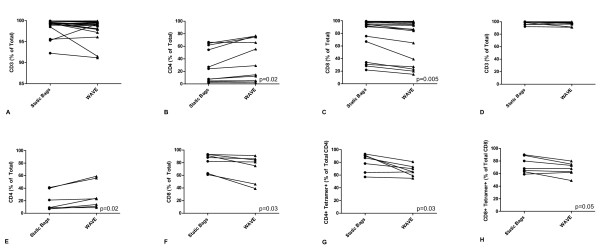
**The WAVE bioreactor produces cultures with greater numbers of CD4+ cells than static bags**. The cell surface expression profile of TIL a) CD3 b) CD4 c) CD8 and genetically modified PBL d) CD3 e) CD4 f) CD8 g) CD4 and Tetramer h) CD8 Tetramer in static bags (circles) and the WAVE bioreactor (triangles).

**Figure 5:**
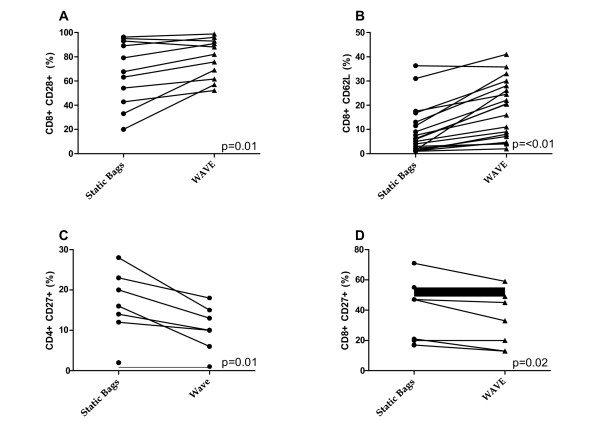
**The WAVE bioreactor and static bags produce cultures with a different phenotypic composition**. A comparison of TIL activation status by cell surface expression of a) CD8 and CD28 b) CD8 and CD62L and genetically modified PBL activation status by cell surface expression of c) CD4 CD27 d) CD8 CD27 in static bags (circles) and the WAVE bioreactor (triangles).

To determine whether the cellular composition of TIL expanded under conditions of constant perfusion would differ from TIL expanded using static bags, the differentiation status of patients' TIL simultaneously expanded in both bioreactors was determined by FACS analysis. The following cell surface markers were evaluated, CD3, CD4, CD8, CD56, CD45RA, CD45RO, CD62L, CCR7, CD27 and CD28 (Figures 4 and 5). Expansion in either static bags or the WAVE bioreactor did not affect the percentage of CD3+ positive TIL or genetically modified PBL (Figure 3a and 3d). Overall WAVE bioreactor expanded TIL had a lower proportion of CD8 positive cells (Figure 4c) and a higher proportion of CD4 positive cells (Figure 4b) than concurrent expansions in static bags (p = 0.005 and p = 0.02 respectively). Interestingly, genetically modified PBL rapidly expanded in the WAVE bioreactor also had a significantly lower percentage of CD8+ cells and a significantly higher percentage of CD4+ cells compared to the expansions carried out in static bags (p = 0.03 and p = 0.02 respectively) (Figure 4e,f). The percentage of CD8 + cells that were tetramer positive between the WAVE bioreactor and static bags appears to decrease, although this does not reach statistical significance. This probably is due to the relatively small sample size (Figure 4h). The WAVE bioreactor had a lower percentage of tetramer + CD4+ cells compared to the static bags, suggesting that the WAVE bioreactor preferentially expanded populations of untransduced cells (Figure 4g).

Phenotypic analysis revealed a higher percentage of CD8^+^CD28^+ ^and CD8^+^CD62L^+ ^cells (Figures 5a,b) in the WAVE bioreactor than in static bags (p = 0.01, p < 0.01 respectively). In contrast, the percentage of both CD4+ and CD8+ positive TCR transduced cells that were also CD27+ was decreased in WAVE bioreactor expanded cells (p = 0.01 and p = 0.02)(Figure 5c, d) while in the CD8+ fraction there was a slight increase in the percentage of CD45RA + cells (data not shown). All other markers that were analyzed, CD3, CD45RO, CCR7 and CD28, did not vary in a statistically significant manner between the two bioreactors.

## Discussion

Initial experiments were performed to optimize the starting cell number, seeding density, day of WAVE bioreactor inoculation and rocking speed. Static bags and the WAVE bioreactor expanded TIL and genetically modified PBL comparably in terms of total cell number, overall fold expansion and viability. Both bioreactors produce cells free of bacterial and fungal contamination, with levels of endotoxin that met or exceeded the requirements of the certificate of analysis. The composition of the final cell product differed between the two bioreactors with the WAVE bioreactor producing a cell product with a higher percentage of CD4+ cells and a lower percentage of CD8+ cells. In addition to differences in the cellular composition of final product there were also differences in the activation status of the cells produced in the two bioreactors, with WAVE bioreactor expanded TIL cultures having a higher percentage of CD8+ CD28+ and CD8+ CD62L positive cells compared to static bag cultures. Genetically modified PBL expanded in the WAVE bioreactor, in contrast, had a lower percentage of CD27+ cells in both the CD4+ and CD8+ compartments than cultures expanded in static bags. The functionality of genetically modified PBL expanded in the two bioreactors was tested in coculture experiments. When cocultures were performed on T2 cells pulsed with cognate peptides for the transduced TCR, WAVE bioreactor expanded genetically modified PBL secreted more IFN-γ (P < 0.05) at three of the peptide concentrations tested. Genetically modified PBL expanded in the two bioreactors were also cocultured with HLA matched and mismatched melanoma cell lines and there was no difference in the amount of IFN-γ secreted. In an ongoing clinical trial both WAVE bioreactor expanded and static bag expanded TIL were able to mediate a significant shrinkage in the tumor volume of a subset of patients; however due to the short follow up period the durability of these responses cannot be assessed.

TIL have been successfully expanded in several styles of bioreactor, including stirred bioreactors [20], hollow fiber bioreactors [21,22], a single pass closed system bioreactor from Aastrom Biosciences [23] as well as in the WAVE bioreactor [24]. Many bioreactors suffer from problems of scalability, having an upper volume limit that prevents the generation of clinically useful cell numbers with a single unit. The WAVE is a closed system bioreactor that has been used extensively in the manufacture of biologics under cGMP compliant conditions [25,26]. The major advantages of the WAVE bioreactor include the small footprint, and the ability to sample the expansion in real time to monitor cell growth and bioreactor conditions. The constant perfusion of media in to the WAVE bioreactor prevents the accumulation of waste products, such as ammonia and lactate, while maintaining the glucose and glutamine levels in an optimal range, allowing TIL/PBL cultures to grow in a reduced volume of culture media at high densities. The reduced volume of patients' cultures greatly expedites the downstream processing prior to infusion, allowing multiple cell products to be harvested per day, using standard blood bank cell processing equipment. The total number of culture manipulations is reduced when expansions are carried out in the WAVE bioreactor, reducing the opportunity for inadvertent operator culture contamination. The closed nature of this system also reduces the requirement for large amounts of clean room space, as media changes can be performed by sterile welding media bags on to the bioreactor.

It is clear that the number and durability of clinical responses is impacted by composition/quality of the infused cell product. Previously, Bessar *et al *noted that melanoma patients who responded to adoptively transferred TIL, received greater absolute numbers of CD8+ cells than non responders enrolled on the same trial [27]. CD8+ cells are the effector cell type responsible for tumor cell destruction and *in vitro*, highly activated CD8+ effector memory cells are the most potent mediators of tumor cell lysis. In mice, the most effective cells upon adoptive transfer are not the most highly activated CD8+ effector memory cell, but a less activated central memory cell type and this appears to relate to the ability of this cell type to persist in the host post transfer [28,29]. Successful treatment with TIL and genetically modified PBL relies on lymphodepletion prior to adoptive transfer, which is believed to reduce endogenous competitors for homeostatic cytokines [30]. Upon transfer cells enter a lymphopenic environment that is enriched for homeostatic cytokines; the less activated central memory like cells express higher levels of receptors for these cytokines than effector memory like cells, and this coupled with their lower rate of apoptosis and higher proliferative potential results in increased engraftment and persistence.

Due to the fact that CD8+ effector cells are our desired cell population the increased representation of CD4+ cells in rapid expansions carried out in the WAVE bioreactor compared to static bags is problematic. The underlying cause of this skewed expansion could relate to differences in the culture microenvironment generated in the two styles of bioreactor. In static bags the cells rest on the lower surface of the bag and there is minimal mixing of the bag contents. In this situation there is extensive cell to cell contact and any secreted factors essential to cell expansion will exist as gradients, with the highest concentration surrounding the cells. Contrast this with the WAVE bioreactor, where the coupling of rocking with media perfusion creates a more homogenous suspension of cells and oxygenated nutrient rich media. In this situation any secreted factors will be diluted by the continual perfusion, while the constant motion will disrupt any gradient formation and reduce the overall time that cells spend in contact with each other. A second possibility relates to the need for a surfactant to be present in the WAVE bioreactor media to protect cells from hydrodynamic/shear force damage. When we attempted to expand TIL in the absence of the surfactant, Pluronic F68, there was significant cell damage, which resulted in a reduction in cell viability. In this situation it was necessary to perform a filtration step prior to harvest to remove the cellular debris, which resulted in further cell loss and introduced an additional step at which the product could become contaminated. To reduce the amount of cell debris, all TIL and genetically modified PBL had their WAVE bioreactor expansions performed in the presence of 0.02% Pluronic F68. In the literature there is some disagreement as to how non-ionic surfactants such as Pluronic F68 protect cells. Murhammer and Goochee [31] have suggested that the pluronic F68 protects by inserting into and altering the physical properties of the plasma membrane. This may alter membrane fluidity and the ability of sensitive cells to respond appropriately in culture, resulting in the observed skewing of expansion in favor of CD4+ cells.

The WAVE bioreactor produces fewer overall CD8+ TIL and genetically modified PBL, which may have an impact on overall response rates. To date we have only performed the final 7 days of rapid expansion in the WAVE bioreactor and it is not clear if this bias would become more pronounced if cultures are used to inoculate the WAVE bioreactor prior to day 7. One solution to the expansion bias in favor of CD4+ cells is to perform CD8+ enrichments on TIL prior to rapid expansion, thus ensuring that higher absolute numbers of CD8+ are available for patient infusion. Of greater concern is the observation that the WAVE bioreactor produced a final cell product with fewer transduced CD4+ and CD8+ cells as assessed by tetramer staining than our standard method using static bags. This suggests that the WAVE bioreactor is preferentially expanding untransduced cells, in particular untransduced CD4+ cells. This produces a cell product that has a reduced representation of cells that are able to specifically target and lyse tumor cells and one would predict this would translate in to poorer clinical outcomes.

Because the activation status of transferred cells also relates to their ability to engraft and clear bulky tumor masses, we assessed the activation status of TIL and genetically modified PBL that underwent concurrent expansion in the WAVE bioreactor and static bags. Cultures that were rapidly expanded in the WAVE bioreactor had a higher percentage of CD8+ CD62L + cells. In addition, the percentage of CD28 positive cells was also elevated in WAVE bioreactor expanded cultures, specifically in the CD8+ cell subset, indicative of these cells having certain characteristics that are associated with greater persistence and efficacy upon transfer in murine models. The WAVE bioreactor therefore appears to be generating CD8+ TIL with desirable characteristics, and this could be exploited by only using CD8+ enriched TIL in rapid expansions to compensate for any preferential expansion of CD4+ cells. Interestingly, genetically modified PBL expanded in the WAVE bioreactor have a more activated phenotype, as indicated by a reduced percentage of CD27 positive cells, than cells expanded in static bags. It is unclear why these two cell products behave differently in the WAVE bioreactor, but, in part, it may be a reflection of the origin of the cells used to initiate the expansions. TIL are isolated from a tumor and are exposed to their cognate antigens. PBL, in contrast, will have a higher proportion of naïve cells. If the WAVE bioreactor is to be used to expand genetically modified PBL the current conditions will have to be modified to ensure that the untransduced cells do not out grow the transduced cells during the rapid expansion. The activation status of these PBL also needs to be investigated further; generally the down regulation of CD27 is associated with a more activated phenotype and a decreased persistence post transfer. For our purposes, the preferential expansion of CD4+ cells is problematic; however, for protocols that utilize a CD4+ based cell product this skewing of expansion could be exploited, such as in the production of Tregs for the treatment of conditions, such as Type1 diabetes and for allogenic transplants [32,33].

We were unable to reliably initiate clinical scale rapid expansions directly in the WAVE bioreactor. The WAVE bioreactor was able to achieve high cell densities, in excess of 1 x10^7 ^cells per ml, after an initial 7 day expansion in T-175 flasks. In a previous report, Sadeghi *et al *[24] performed concurrent rapid expansions of TILs from 4 donors starting directly in the WAVE bioreactor and static bags and found similar fold expansions in the two bioreactors (228 and 72 fold expansion in the WAVE and static bags respectively). However, their reported total fold expansion was too low to meet our clinical needs. In contrast to our findings, they observed no phenotypic or functional variation between the two bioreactors. There are significant differences in the media compositions and methodologies used in these two studies, which makes direct comparison of the results impossible.

There are some disadvantages with the WAVE bioreactor including a difficult transition from research scale expansions, where the initial cell number and seeding density are determined, along with the day of WAVE inoculation and rocking speed, to full scale clinical expansions. In addition to the WAVE bioreactors themselves it is necessary to purchase additional ancillary equipment. This, along with the inherent complexities of a system that depends on constant motion, such as a predisposition to electrical and mechanical failure, means that there is a need for multiple bioreactors to expand a single patient's cells. In order to exclusively adopt the WAVE bioreactor as the sole platform for rapid expansions, therefore requires a substantial initial investment, which may be beyond the means of a small cell production facility that produces only a minimal number of cell products; however, the WAVE bioreactor is ideal for larger scale manufacturing facilities, where multiple cell products need to manufactured concurrently. We have identified alternative bioreactors which don't require an additional expenditure on ancillary equipment, as they utilize equipment and monitoring systems that are standard to most cell production facilities [34].

## Conclusions

The Surgery Branch of the National Cancer Institute has had success treating patients with cells expanded in static bags and it is clear that cells expanded in this style of bioreactor can mediate regressions in patients with advanced, bulky, metastatic melanoma [9]. We have evaluated the WAVE bioreactor, in an attempt to create a simplified, GMP compliant clinical expansion protocol. Differences in the phenotype of cells generated in this bioreactor compared to our standard methodology could impact the frequency or durability of clinical responses, but these outcomes will need to be evaluated in a clinical trial. The reduction in labor required to maintain cultures in the WAVE bioreactor, coupled with the closed nature of this system, suggests that this system can be a suitable alternative to the static gas permeable bags.

## Abbreviations

TIL: Tumor infiltrating lymphocyte; ACT: Adoptive cell transfer therapy; REP: Rapid expansion protocol; cGMP: Current good manufacturing practices.

## Competing interests

The authors declare that they have no competing interests.

## Authors' contributions

RPTS performed T cell rapid expansions, data analysis and drafted the manuscript. LD performed all FACS analyses. MRP developed the T cell rapid expansion protocol in the WAVE bioreactor. SAR conceived and participated in the design of study. MED designed and coordinated the study and drafted the manuscript. All authors read and approved the final manuscript.

## References

[B1] Balch CM Gershenwald JE Soong SJ Thompson JF Atkins MB Byrd DR Final version of 2009 AJCC melanoma staging and classification J Clin Oncol 2009 27 6199 6206 10.1200/JCO.2009.23.4799 19917835 PMC2793035

[B2] Middleton MR Grob JJ Aaronson N Fierlbeck G Tilgen W Seiter S Randomized phase III study of temozolomide versus dacarbazine in the treatment of patients with advanced metastatic malignant melanoma J Clin Oncol 2000 18 158 166 10623706 10.1200/JCO.2000.18.1.158

[B3] Atkins MB Lotze MT Dutcher JP Fisher RI Weiss G Margolin K High-dose recombinant interleukin 2 therapy for patients with metastatic melanoma: analysis of 270 patients treated between 1985 and 1993 J Clin Oncol 1999 17 2105 2116 10561265 10.1200/JCO.1999.17.7.2105

[B4] Hodi FS O'Day SJ McDermott DF Weber RW Sosman JA Haanen JB Improved survival with ipilimumab in patients with metastatic melanoma N Engl J Med 2010 363 711 723 10.1056/NEJMoa1003466 20525992 PMC3549297

[B5] Atkins MB Kunkel L Sznol M Rosenberg SA High-dose recombinant interleukin-2 therapy in patients with metastatic melanoma: long-term survival update Cancer J Sci Am 2000 6 Suppl 1 S11 S14 10685652

[B6] Flaherty KT Puzanov I Kim KB Ribas A McArthur GA Sosman JA Inhibition of mutated, activated BRAF in metastatic melanoma N Engl J Med 2010 363 809 819 10.1056/NEJMoa1002011 20818844 PMC3724529

[B7] Rosenberg SA Restifo NP Yang JC Morgan RA Dudley ME Adoptive cell transfer: a clinical path to effective cancer immunotherapy Nat Rev Cancer 2008 8 299 308 10.1038/nrc2355 18354418 PMC2553205

[B8] Dudley ME Wunderlich JR Shelton TE Even J Rosenberg SA Generation of tumor-infiltrating lymphocyte cultures for use in adoptive transfer therapy for melanoma patients J Immunother 2003 26 332 342 10.1097/00002371-200307000-00005 12843795 PMC2305721

[B9] Dudley ME Gross CA Langhan MM Garcia MR Sherry RM Yang JC CD8+ enriched "young" tumor infiltrating lymphocytes can mediate regression of metastatic melanoma Clin Cancer Res 2010 16 6122 6131 10.1158/1078-0432.CCR-10-1297 20668005 PMC2978753

[B10] Rosenberg SA Yang JC Sherry RM Kammula US Hughes MS Phan GQ Durable Complete Responses in Heavily Pretreted Patients with Metastatic Melanoma Using T Cell Transfer Immunotherapy Clin Cancer Res 2011 13 4550 7 21498393 10.1158/1078-0432.CCR-11-0116 PMC3131487

[B11] Morgan RA Dudley ME Rosenberg SA Adoptive cell therapy: genetic modification to redirect effector cell specificity Cancer J 2010 16 336 341 10.1097/PPO.0b013e3181eb3879 20693844 PMC6348476

[B12] Robbins PF Morgan RA Feldman SA Yang JC Sherry RM Dudley ME Tumor regression in patients with metastatic synovial cell sarcoma and melanoma using genetically engineered lymphocytes reactive with NY-ESO-1 J Clin Oncol 2011 29 917 924 10.1200/JCO.2010.32.2537 21282551 PMC3068063

[B13] Kochenderfer JN Wilson WH Janik JE Dudley ME Stetler-Stevenson M Feldman SA Eradication of B-lineage cells and regression of lymphoma in a patient treated with autologous T cells genetically engineered to recognize CD19 Blood 2010 116 4099 4102 10.1182/blood-2010-04-281931 20668228 PMC2993617

[B14] Parkhurst MR Yang JC Langan RC Dudley ME Nathan DA Feldman SA T cells targeting carcinoembryonic antigen can mediate regression of metastatic colorectal cancer but induce severe transient colitis Mol Ther 2011 19 620 626 10.1038/mt.2010.272 21157437 PMC3048186

[B15] Prieto PA Durflinger KH Wunderlich JR Rosenberg SA Dudley ME Enrichment of CD8+ cells from melanoma tumor-infiltrating lymphocyte cultures reveals tumor reactivity for use in adoptive cell therapy J Immunother 2010 33 547 556 10.1097/CJI.0b013e3181d367bd 20463593 PMC6309789

[B16] Tran CA Manufacturing of large numbers of patient-specific T cells for adoptive immunotherapy: an approach to improving product safety, composition, and production capacity 2007 10.1097/CJI.0b013e318052e1f4 17667528

[B17] Tran KQ Minimally cultured tumor-infiltrating lymphocytes display optimal characteristics for adoptive cell therapy 2008 10.1097/CJI.0b013e31818403d5 PMC2614999 18779745

[B18] Johnson LA Morgan RA Dudley ME Cassard L Yang JC Hughes MS Gene therapy with human and mouse T-cell receptors mediates cancer regression and targets normal tissues expressing cognate antigen Blood 2009 114 535 546 10.1182/blood-2009-03-211714 19451549 PMC2929689

[B19] Morgan RA Dudley ME Wunderlich JR Hughes MS Yang JC Sherry RM Cancer regression in patients after transfer of genetically engineered lymphocytes Science 2006 314 126 129 10.1126/science.1129003 16946036 PMC2267026

[B20] Carswell KS Papoutsakis ET Culture of human T cells in stirred bioreactors for cellular immunotherapy applications: shear, proliferation, and the IL-2 receptor Biotechnol Bioeng 2000 68 328 338 10.1002/(SICI)1097-0290(20000505)68:3<328::AID-BIT11>3.0.CO;2-V 10745201

[B21] Hami LS Green C Leshinsky N Markham E Miller K Craig S GMP production and testing of Xcellerated T Cells for the treatment of patients with CLL Cytotherapy 2004 6 554 562 10.1080/14653240410005348 15764021

[B22] Levine BL T lymphocyte engineering ex vivo for cancer and infectious disease Expert Opin Biol Ther 2008 8 475 489 10.1517/14712598.8.4.475 18352851

[B23] Klapper JA Thomasian AA Smith DM Gorgas GC Wunderlich JR Smith FO Single-pass, closed-system rapid expansion of lymphocyte cultures for adoptive cell therapy J Immunol Methods 2009 345 90 99 10.1016/j.jim.2009.04.009 19389403 PMC2700005

[B24] Sadeghi A Pauler L Anneren C Friberg A Brandhorst D Korsgren O Large-scale bioreactor expansion of tumor-infiltrating lymphocytes J Immunol Methods 2011 364 94 100 10.1016/j.jim.2010.11.007 21111743

[B25] Hollyman D Stefanski J Przybylowski M Bartido S Borquez-Ojeda O Taylor C Manufacturing validation of biologically functional T cells targeted to CD19 antigen for autologous adoptive cell therapy J Immunother 2009 32 169 180 10.1097/CJI.0b013e318194a6e8 19238016 PMC2683970

[B26] Eibl R Eibl D Application of Disposable Bag-Bioreactors in Tissue Engineering and for the Production of Therapeutic Agents Adv Biochem Eng Biotechnol 2009 112 183 207 19290502 10.1007/978-3-540-69357-4_8

[B27] Besser MJ Shapira-Frommer R Treves AJ Zippel D Itzhaki O Hershkovitz L Clinical Responses in a Phase II Study Using Adoptive Transfer of Short-term Cultured Tumor Infiltration Lymphocytes in Metastatic Melanoma Patients Clin Cancer Res 2010 16 2646 2655 10.1158/1078-0432.CCR-10-0041 20406835

[B28] Gattinoni L Klebanoff CA Palmer DC Wrzesinski C Kerstann K Yu Z Acquisition of full effector function in vitro paradoxically impairs the in vivo antitumor efficacy of adoptively transferred CD8+ T cells J Clin Invest 2005 115 1616 1626 10.1172/JCI24480 15931392 PMC1137001

[B29] Klebanoff CA Gattinoni L Torabi-Parizi P Kerstann K Cardones AR Finkelstein SE Central memory self/tumor-reactive CD8+ T cells confer superior antitumor immunity compared with effector memory T cells Proc Natl Acad Sci USA 2005 102 9571 9576 10.1073/pnas.0503726102 15980149 PMC1172264

[B30] Gattinoni L Finkelstein SE Klebanoff CA Antony PA Palmer DC Spiess PJ Removal of homeostatic cytokine sinks by lymphodepletion enhances the efficacy of adoptively transferred tumor-specific CD8+ T cells J Exp Med 2005 202 907 912 10.1084/jem.20050732 16203864 PMC1397916

[B31] Murhammer DW Goochee CF Sparged animal cell bioreactors: mechanism of cell damage and Pluronic F-68 protection Biotechnol Prog 1990 6 391 397 10.1021/bp00005a012 1366875

[B32] Bacchetta R Gregori S Barbarella L Roncarolo MG Cell therapy with human T regulatory type 1 cells in allogeneic transplantations Immunotherapy 2011 3 2721174555

[B33] Filippi C Bresson D von Herrath M von HM: Antigen-specific induction of regulatory T cells for type 1 diabetes therapy Int Rev Immunol 2005 24 341 360 10.1080/08830180500371116 16318986

[B34] Vera JF Brenner LJ Gerdemann U Ngo MC Sili U Liu H Accelerated production of antigen-specific T cells for preclinical and clinical applications using gas-permeable rapid expansion cultureware (G-Rex) J Immunother 2010 33 305 315 10.1097/CJI.0b013e3181c0c3cb 20445351 PMC2946348

